# Personal income perception and the risk of metabolic dysfunction-associated steatotic liver disease

**DOI:** 10.3389/fnut.2026.1739165

**Published:** 2026-03-05

**Authors:** Gianluigi Casimo, Rossella Donghia, Rossella Tatoli, Caterina Bonfiglio, Gianluigi Giannelli

**Affiliations:** 1 Data Science Unit, National Institute of Gastroenterology-IRCCS “Saverio de Bellis”, Castellana Grotte, Italy; 2 Scientific Direction, National Institute of Gastroenterology-IRCCS “Saverio de Bellis”, Castellana Grotte, Italy

**Keywords:** demographic, food insecurity, income, MASLD, personal assessment, socioeconomic

## Abstract

**Background:**

In recent years, researchers have discovered that the development of MASLD disease does not solely depend on medical conditions and dietary behaviors, but is also influenced by sociodemographic and economic factors (e.g., education and income). This study aims to analyse the association between personal satisfaction with one’s income and the development of MASLD disease in a cohort from the southeast of Italy.

**Methods:**

The analysis involved 1,297 participants from the NUTRIEP project, who completed a questionnaire about their medical history, dietary behaviors, and socioeconomic background. The participants’ personal assessment of their income was categorised into five levels from “Totally insufficient” to “Good”.

**Results:**

The logistic regression model revealed a significant protective effect of personal satisfaction regarding income in relation to the development of MASLD. The Odds Ratios of participants were OR = 0.55 (*p* = 0.186, 95% C.I. 0.23; 1.33), OR = 0.40 (*p* < 0.05, 95% C.I. 0.17; 0.92), OR = 0.22 (*p* < 0.05, 95% C.I. 0.08; 0.61), and OR = 0.17 (*p* < 0.05, 95% C.I. 0.05; 0.55) across the categories ranging from “Just Sufficient” to “Sufficient,” “More than Sufficient,” and “Good” satisfaction with income.

**Conclusion:**

This research confirms the association between the development of MASLD and income satisfaction, supporting the notion that this condition is linked to the social context of those affected. The analysis also highlighted dietary behaviors related to income and personal perception. Less satisfied individuals exhibited a higher kcal intake and lower rMED scores, indicating a greater consumption of low-quality energy-dense foods. Furthermore, this research emphasizes the widening wage disparity among social classes, which could threaten to the public health.

## 1 Introduction

An increase in chronic liver diseases within the population has recently become a great concern for health organisations. In 2017, these diseases caused 1.32 million deaths worldwide (1, 2).

In particular, recent years have seen a heightened focus, on Metabolic Dysfunction-Associated Steatotic Liver Disease (MASLD), due to its rapid spread in many regions, making it the most prevalent cause of chronic liver diseases (3, 4). In fact, its prevalence has risen from 25.3 to 38.2% over the past 30 years (3) globally, totaling approximately 1.66 billion cases (3).

The definition of MASLD is relatively new; it was introduced in 2023 at the Delphi process (5) to replace the previous NAFLD (Non-Alcoholic Fatty Liver Disease). This change was made to broaden the approach to the disease to a multidisciplinary level (5) reflecting the growing concern for public health. With the new denomination, it is no longer just a relegated liver disease but also recognized as a risk factor for several other extrahepatic conditions, including cardiovascular disease (CVD) and chronic kidney disease (CKD) (3).

MASLD is defined as the intrahepatic accumulation of triglycerides, which can be detected through imaging or biopsy, in the absence of significant alcohol consumption and in association with at least one of the following metabolic alterations: overweight or obesity (BMI ≥ 25 kg/m^2^), elevated fasting blood glucose levels (≥ 100 mg/dL), hypertension (blood pressure ≥ 130/85 mmHg), dyslipidemia (plasma triglycerides ≥ 150 mg/dL), or low levels of HDL cholesterol (40 mg/dL for men and < 50 mg/dL for women) (6).

In trecent years, researchers have not only begun to consider clinical histories and dietary behaviors as risk factors for the development of MASLD, but also sociological factors, such as education (7) and income (8, 9) as key factors that can influence the onset of this disease.

As Western societies entered a recession phase (10, 11) over the past few decades, the concerns about family income and resulting food insecurity (8–10, 12–16) have increased. Bruenin et al. (10) found that as poverty levels rise among US families, the percentage of energy-dense food in their diets also increases, contributing to a rise in metabolic disorders, particularly obesity. This phenomenon has been referred to as the “hunger-obesity paradox” (10, 12, 13). Therefore, the current economic situation may serve as a catalyst for the global spread of MASLD.

Building on this understanding, our study analyzes the potential association between personal assessment and satisfaction with financial wealth and the development of MASLD in a cohort from Southern Italy. To date, there are no studies in the scientific literature that examine this issue in a population comparable to ours.

## 2 Materials and methods

### 2.1 Study population

The NUTRIHEP study is a cohort established in 2005–2006, using a systematic random sample of Putignano Primary Care Physicians’ attendees over 18 (17). We used general practitioners’ (GP) registers instead of census data because no significant differences were found in age and gender distribution between the general population of Putignano and the GP records. From 2015 to 2018, all NUTRIHEP participants were recalled for the first follow-up; 1,426 responded and followed the same protocol as at enrolment. All participants provided informed consent after being fully informed about their medical data to be studied. The study employed a cross-sectional design, focusing only on the follow-up measurement. It was approved by the Ethical Committee of the Minister of Health (DDG-CE-792/2014, 14 February 2014).

### 2.2 Data collection

During follow-up visits, participants completed all assessments outlined in the protocol. Trained physicians and/or nutritionists interviewed them to collect data on sociodemographic factors, health status, personal history, and lifestyle habits. This covered tobacco use history, diet, educational level (18), work profile (19), and marital status.

Weight and height were measured with participants dressed in underclothing and without shoes. Weights were recorded to the nearest 0.1 kg using an electronic balance (SECA^©^), and height was measured to the nearest 1 cm with a wall-mounted stadiometer (SECA^©^). Blood pressure (BP) was obtained following international guidelines (20, 21), with the average of three readings calculated. Participants completed the EPIC food questionnaire to provide information about their dietary habits (22, 23). Blood tests included fasting serum glucose (FSG), fasting insulin (FI), glycated hemoglobin (HbA1c), triglycerides, total cholesterol, low density lipoprotein (LDL-C), high density lipoprotein (HDL-C), aspartate aminotransferase (AST), alanine aminotransferase (ALT), gamma-glutamyl transferase (GGT), ferritin, and high-sensitivity C-reactive protein, analysed on the COBAS 8000 autoanalyser (ROCHE Diagnostics SPA, Monza, Italy).

Insulin resistance was estimated using the Homeostasis Model Assessment of Insulin Resistance (HOMA-IR) (24), calculated by the following formula:


$$\text{HOMA}-\mathrm{IR}=\frac{\mathrm{FSG}\;(\;\mathrm{mg}/\mathrm{dL}\;)\times \mathrm{FI}\;(\;\;\mathrm{\mu IU}/\mathrm{mL}\;\;)}{405}$$

All subjects underwent standardized ultrasound examinations with a Hitachi H21 Vision (Hitachi Medical Corporation, Tokyo, Japan). The liver parenchyma was examined using a 3.5 MHz transducer. A scoring system was used to provide a semi-quantitative assessment of hepatic fat content. The extent of hepatic fat infiltration was evaluated based on liver echotexture, hepatic echo penetration, clarity of hepatic blood vessels, and differentiation of the hepatic diaphragm in echo amplitude (25) (Supplementary Table S1, S2).

The European Prospective Investigation into Cancer and Nutrition (EPIC) Food Frequency Questionnaire (FFQ) (26) was used to record typical food consumption at the start of the study. Participants completed the EPIC questionnaire themselves and returned it after one week. Nutritionists checked all responses for accuracy and entered the data into a specialized online program. Afterward, the nutritional information was converted into micro- and macro-nutrients.

### 2.3 Outcome assessment

The MASLD definition is based on hepatic steatosis plus at least one cardiometabolic risk factor: (1) BMI > 25 kg/m^2^ or waist circumference >94 cm in men and >80 cm in women; (2) fasting serum glucose ≥ 100 mg/dL (≥5.6 mmol/L), two-hour post-load glucose ≥ 140 mg/dL (≥7.8 mmol/L), HbA1c ≥ 5.7%, or specific drug treatment; (3) blood pressure ≥ 130/85 mmHg or in specific drug treatment; (4) plasma triglycerides ≥ 150 mg/dL (≥1.70 mmol/L) or in specific drug treatment; (5) plasma HDL cholesterol < 40 mg/dL (<1.0 mmol/L) for men and <50 mg/dL (<1.3 mmol/L) for women, or in specific drug treatment. The definition also limits alcohol intake—20–50 g/day for women and 30–60 g/day for men—focusing on steatosis, similar to NAFLD (6). Additionally, other liver diseases, like MASLD + HCV, are excluded to prevent interference with the disease’s natural progression (27).

### 2.4 Exposure variable

During the interview some socioeconomic informations were asked to the NUTRIEP subjects, in particular concerning their personal assessment of the family income. So they expressed an opinion based on how their income fulfil their lifestyle, on a scale from 1 to 5, where 1 was equal to “Totally Insufficient,” the second class was equal to “Just Sufficient,” the third was “Sufficient,” 4 was equal to “More than Sufficient” and, finally, 5 was equal to “Good”.

### 2.5 Confounding variables

Covariates were selected based on previous studies and clinical and statistical judgment on their potential association with MASLD. After evaluating potential collinearity, we included the following sociodemographic covariates: age (as a categorical variable about under or over 65 years), gender, work, marital status, personal income and house location. Furthermore some lifestyle variable were included: total of kcal taken in a day and the rMED score. Lastly the HOMA variable was also included as confounding. These variable were chosen in order to better estimate not only the socioeconomic wealth of each patient, but also their physiological and nutritional wellness.

In order not to run into collinearity problems, variables that fall under the definition of MASLD were excluded: BMI, Waist, Fasting Glucose, Triglycerides, Blood Pressure, HDL cholesterol, and HbA1c.

### 2.6 Statistical analysis

The individuals characteristics are reported as means and standard deviations (M ± SD) or medians and interquartile ranges for continuous variables and as frequencies and percentages (%) for categorical variables. We used the Wilcoxon signed-rank test for continuous variables to compare two groups or the Kruskal-Wallis test for more than two groups; for categorical variables, we used the χ2 or the Fisher test. We fitted a logistic regression model to estimate odds ratios (ORs) and 95% Confidence Intervals (CIs) with MASLD as the outcome variable and the personal assessment of the family income as predictor.

Odds Ratios (OR) are utilised to estimate the odds of an event occurring in one group compared to another, and they are frequently employed to assess the risk of a negative outcome associated with a treatment or intervention. An OR of 1 indicates no difference between the groups, an OR greater than 1 suggests an increased risk in the exposure group, and an OR of less than 1 indicates a decreased risk (28). The model was adjusted for the following variables: age (under or over 65 years), gender, work, marital status, personal income, house location, rMED, total daily kcal assumption and HOMA values, and the estimated coefficients were transformed into odds ratio (OR), using the “Totally Insufficient” class as reference.

Initially, confounding variables were selected from the existing literature. Subsequently, the minimum absolute reduction and selection (LASSO) (29) was employed to decrease the number of candidate predictors and identify those most useful for model construction. In addition, the variance inflation factor (VIF) was also evaluated to check multicollinearity, and confounders with VIF > 5 were discarded (Supplementary Table S3) (30). The two-tailed probability level was set at 0.05 to test the null hypothesis of non-association.

The analyses were conducted with StataCorp 2025 Stata Statistical Software: Release 18.0 (College Station, TX, United States: StataCorp LLC.).

## 3 Results

Table 1 presents the distribution of sociodemgraphic parameters for NUTRIEP subjects based on their self assessment of family income. An analysis of the annual family income reveal an approximately linear correlation between the declared income and the subjects’ assessments (*p* < 0.001). However, a small group of participants opted not to disclose their family income, probably due to a social taboo in western cultures linked to the social implication of this topic (31). Most participants who identified as poor also reported a lower assessment of their economic wellness, while those identified as wealthy reported the opposite.

**Table 1:** Socio-demographic and clinical parameters of NUTRIEP subjects, based on personal assessment of family income.

	Personal assessment of family income						
Parameters*	Totally insufficient	Just sufficient	Sufficient	More than sufficient	Good	Total	*p*-value^†^
N. participants (%)	27 (2.10)	167 (12.90)	1,019 (78.60)	64 (4.90)	20 (1.50)	1,297 (100.00)	
Age (SD)	57.51 (14.16)	52.88 (14.10)	54.88 (14.34)	48.16 (13.08)	53.89 (16.17)	54.33 (14.34)	0.003
MASLD (%)							0.023^
No	10 (37.00)	81 (48.50)	521 (51.10)	44 (68.80)	12 (60.00)	668 (51.50)	
Yes	17 (63.00)	86 (51.50)	498 (48.90)	20 (31.20)	8 (40.00)	629 (48.50)	
Gender (%)							0.142^
Female	12 (44.40)	106 (63.50)	581 (57.00)	37 (57.80)	8 (40.00)	744 (57.40)	
Male	15 (55.60)	61 (36.50)	438 (43.00)	27 (42.20)	12 (60.00)	553 (42.60)	
Annual income (€) (%)							<0.001^
<5,000	2 (7.40)	0 (0.00)	1 (0.10)	0 (0.00)	0 (0.00)	3 (0.20)	
5,000–10,000	7 (25.90)	25 (15.00)	11 (1.10)	0 (0.00)	0 (0.00)	43 (3.30)	
10,000–20,000	12 (44.40)	85 (50.90)	330 (32.40)	2 (3.10)	0 (0.00)	429 (33.10)	
20,000–30,000	6 (22.20)	38 (22.80)	444 (43.60)	17 (26.60)	1 (5.00)	506 (39.00)	
30,000–40,000	0 (0.00)	5 (3.00)	115 (11.30)	26 (40.60)	2 (10.00)	148 (11.40)	
40,000–50,000	0 (0.00)	0 (0.00)	38 (3.70)	12 (18.80)	6 (30.00)	56 (4.30)	
>50,000	0 (0.00)	0 (0.00)	8 (0.80)	4 (6.20)	9 (45.00)	21 (1.60)	
No answer	0 (0.00)	0 (0.00)	1 (0.10)	0 (0.00)	0 (0.00)	1 (0.10)	
Do not know	0 (0.00)	14 (8.40)	71 (7.00)	3 (4.70)	2 (10.00)	90 (6.90)	
Work (%)							<0.001^
Managers & professionals	0 (0.00)	12 (7.20)	76 (7.50)	10 (15.60)	4 (20.00)	102 (7.90)	
Craft, agricultural and sales workers	7 (25.90)	47 (28.10)	373 (36.60)	32 (50.00)	10 (50.00)	469 (36.20)	
Elementary Occupations	4 (14.80)	40 (24.00)	135 (13.20)	6 (9.40)	0 (0.00)	185 (14.30)	
Housewife	7 (25.90)	15 (9.00)	114 (11.20)	5 (7.80)	0 (0.00)	141 (10.90)	
Pensioners	7 (25.90)	34 (20.40)	270 (26.50)	9 (14.10)	5 (25.00)	325 (25.10)	
Jobless	2 (7.40)	19 (11.40)	51 (5.00)	2 (3.10)	1 (5.00)	75 (5.80)	
Marital status (%)							0.080^
Single	2 (7.40)	22 (13.20)	145 (14.20)	10 (15.60)	2 (10.00)	181 (14.00)	
Married or living together	24 (88.90)	130 (77.80)	810 (79.50)	54 (84.40)	16 (80.00)	1,034 (79.70)	
Separated or divorced	1 (3.70)	4 (2.40)	21 (2.10)	0 (0.00)	2 (10.00)	28 (2.20)	
Widower	0 (0.00)	11 (6.60)	43 (4.20)	0 (0.00)	0 (0.00)	54 (4.20)	
House location (%)							0.091^
Downtown	11 (40.70)	74 (44.30)	353 (38.30)	30 (46.90)	10 (50.00)	478 (39.80)	
Periphery	10 (37.00)	62 (37.10)	440 (47.70)	29 (45.30)	9 (45.00)	550 (45.80)	
Countryside	6 (22.20)	31 (18.60)	129 (14.00)	5 (7.80)	1 (5.00)	172 (14.30)	
HOMA (SD)	1.73 (1.21)	1.74 (1.26)	1.94 (2.03)	1.52 (1.09)	1.96 (1.49)	1.89 (1.88)	0.197
BMI (SD)	29.13 (5.84)	28.10 (5.18)	27.60 (5.04)	26.01 (4.40)	25.53 (3.57)	27.58 (5.05)	0.005
Kcal (x day) (SD)	2,468.92 (928.38)	2,129.26 (813.56)	2,034.86 (734.92)	2,095.68 (731.79)	1,854.01 (593.56)	2,056.26 (750.22)	0.076
rMED score (%)							0.120^
Low	10 (37.00)	53 (31.70)	280 (27.50)	17 (26.60)	5 (25.00)	365 (28.10)	
Medium	15 (55.60)	95 (56.90)	555 (54.50)	29 (45.30)	11 (55.00)	705 (54.40)	
High	2 (7.40)	19 (11.40)	184 (18.10)	18 (28.10)	4 (20.00)	227 (17.50)	

*Mean and standard deviation (M ± SD) for continuous variables and frequency and percentage (%) for categorical variables. ^†^Kruskal–Wallis rank test; ^Chi-square or Fisher test when necessary. MASLD, Metabolic Dysfunction-associated Steatotic Liver Disease; HOMA, homeostatic model assessment of insulin resistance; BMI, body mass index; rMED, Relative Mediterranean Diet.

The age means remain within a narrow range around the total population’s mean (*p* < 0.05), suggesting a homogenous age distribution across the different assessment classes. In contrast, the age distribution sorted by the presence of MASLD disease (Table 2) shows that those affected tend to be older (*p* < 0.001). This is evidenced by a higher percentage of sick individuals among the over 65 age group (69.9%, 239 out of 342) compared to 40.8% (390 out of 955) among those under 65 (*p* < 0.001). Furthermore, pensioners are the most affected group (34.2%, 215 out of 629), suggesting that the disease ha a greater impact on people who are older and more likely to be sedentary.

**Table 2:** Socio-demographic and clinical parameters of NUTRIEP subjects, based on MASLD presence (Y/N).

	MASLD			
Parameters*	*N*	Y	Total	*p-*value^†^
N. participants (%)	668 (51.50)	629 (48.50)	1,297 (100.00)	
Age (SD)	49.24 (13.80)	59.74 (12.86)	54.33 (14.34)	<0.001
Age over 65 years (%)				<0.001^
No	565 (84.60)	390 (62.00)	955 (73.60)	
Yes	103 (15.40)	239 (38.00)	342 (26.40)	
Gender (%)				<0.001^
F	417 (62.40)	327 (52.00)	744 (57.40)	
M	251 (37.60)	302 (48.00)	553 (42.60)	
Annual income (€) (%)				0.004^
<5,000	2 (0.30)	1 (0.20)	3 (0.20)	
5,000–10,000	17 (2.50)	26 (4.10)	43 (3.30)	
10,000–20,000	201 (30.10)	228 (36.20)	429 (33.10)	
20,000–30,000	277 (41.50)	229 (36.40)	506 (39.00)	
30,000–40,000	86 (12.90)	62 (9.90)	148 (11.40)	
40,000–50,000	38 (5.70)	18 (2.90)	56 (4.30)	
>50,000	9 (1.30)	12 (1.90)	21 (1.60)	
No answer	0 (0.00)	1 (0.20)	1 (0.10)	
Do not know	38 (5.70)	52 (8.30)	90 (6.90)	
Work (%)				<0.001^
Managers & professionals	57 (8.50)	45 (7.20)	102 (7.90)	
Craft, agricultural and sales workers	285 (42.70)	184 (29.30)	469 (36.20)	
Elementary occupations	93 (13.90)	92 (14.60)	185 (14.30)	
Housewife	74 (11.10)	67 (10.70)	141 (10.90)	
Pensioners	110 (16.50)	215 (34.20)	325 (25.10)	
Jobless	49 (7.30)	26 (4.10)	75 (5.80)	
Marital status (%)				<0.001^
Single	115 (17.20)	66 (10.50)	181 (14.00)	
Married or living together	519 (77.70)	515 (81.90)	1,034 (79.70)	
Separated or divorced	20 (3.00)	8 (1.30)	28 (2.20)	
Widower	14 (2.10)	40 (6.40)	54 (4.20)	
House location (%)				0.860^
Downtown	244 (39.40)	234 (40.30)	478 (39.80)	
Periphery	284 (45.80)	266 (45.90)	550 (45.80)	
Countryside	92 (14.80)	80 (13.80)	172 (14.30)	
HOMA (SD)	1.33 (0.90)	2.43 (2.38)	1.89 (1.88)	<0.001
BMI (SD)	25.04 (3.59)	30.29 (4.97)	27.58 (5.05)	<0.001
Kcal (x day) (SD)	2,100.33 (724.88)	2,009.46 (774.05)	2,056.26 (750.22)	0.002
rMED score (%)				0.460^
Low	196 (29.30)	169 (26.90)	365 (28.10)	
Medium	362 (54.20)	343 (54.50)	705 (54.40)	
High	110 (16.50)	117 (18.60)	227 (17.50)	

*Mean and standard deviation (M ± SD) for continuous variables and frequency and percentage (%) for categorical variables. ^†^Wilcoxon signed-rank test; ^Chi-square or Fisher test when necessary. MASLD, Metabolic Dysfunction-associated Steatotic Liver Disease; HOMA, homeostatic model assessment of insulin resistance; BMI, body mass index; rMED, Relative Mediterranean Diet.

The analysis indicates that, although not statistically significant (*p* = 0.142), personal assessments of family income reveal a greater tendency for women to express dissatisfaction compared to men. Conversely, men tend to have a more favorable view of their income. Moreover, the quasi-linear correlation between the personal assessments and actual income suggests that men generally have slightly higher incomes, highlighting a persistent wage disparity in our society.

It is possible to associate personal assessments also with the subjects’ eating habits; there is a clear correlation between these assessments and the daily kilocalorie consumption of the subjects (*p* = 0.076). Subjects who are more satisfied with their financial situation tend to consume fewer kilocalories (e.g., 1,854.01 kcal for the “Good” class, 2,468.92 kcal for the “Totally Insufficient”). This may be attributed to wealthier people having easier access to healthier, lower-calorie food such as vegetables. In fact, wealthier subjects also tend to have a better rMED score indicating that their daily diets align more closely with a Mediterranean diet than those of poorer individuals.

These observations regarding the relationship between the assessments and eating habits confirm the clear association shown with MASLD disease (*p* < 0.05). This is evident when comparing the distribution of subjects across the assessment classes (Table 1). In the “Totally Insufficient” and “Just Sufficient” classes, MASLD-positive people are in majority (63.0 and 51.5%). However, in the other three classes, the trend shifts, with the majority consisting of MASLD-negative individuals (51.5, 68.8 and 60.0%). This correlation is also corroborated by the MASLD trend observed across certain classes of family annual income (*p* < 0.05). Specifically, there is a decrease in the percentage of ill individuals, moving from the “5,000–10,000” class to the “40,000–50,000” bracket, with rates declining from 60.5% (26 out of 43) to 32.1% (18 out of 56). Additionally, a notable trend can be observed in the BMI distribution, which is higher among dissatisfied individuals and decreases as satisfaction levels improve; this pattern is consistent with the distribution of MALSD-positive and -negative data.

The association between MASLD and personal income was evaluated using the logistic model presented in Table 3, which was adjusted for several covariates, as previously described in the statistical analysis section.

**Table 3:** Logistic regression analysis of MASLD (yes/no) in NUTRIEP subjects on personal assessment of family income, “totally insufficient” class as reference.

MASLD	Odds ratio	S.E. (OR)	*p*-value	95% C.I.
Personal assessment				
Totally insufficient	1.00	--	--	--
Just sufficient	0.55	0.25	0.186	0.23; 1.33
Sufficient	0.40	0.17	0.031	0.17; 0.92
More than sufficient	0.22	0.11	0.004	0.08; 0.61
Good	0.17	0.10	0.003	0.05; 0.55

MASLD, metabolic dysfunction-associated steatotic liver disease; se (OR), standard error of OR; 95% C.I., confidential interval at 95%. Model adjusted for gender, age (>65 years vs. <65 years), annual family income, work status, marital status, house location, daily kilocalories intake, rMED score, HOMA.

The probability of developing MASLD disease is already reduced by half in the “Just Sufficient” class (O.R. = 0.55). However, this finding is not statistically significant (*p* = 0.186) probably due to a small difference in personal assessment between the two classes. The protective effect of socioeconomic-wellness increases those categorized as “Sufficient” (O.R. = 0.40, *p* = 0.031, 95% C.I. = 0.17 to 0.92). This effect reaches ~ 80% more protection for individuals with a “More than Sufficient” socioeconomic status (O.R. = 0.22, *p* = 0.004, 95% C.I. = 0.08 to 0.61), compared to the most dissatisfied class. Finally, the group classified as “Good” demonstrates the highest level of protection, with an odds ratio of 0.17 (*p* = 0.003, 95% C.I. = 0.05 to 0.55), confirming the increasing protective effect of socioeconomic wellness against this liver disease.

## 4 Discussion

The analysis of the association between MASLD and personal assessments of family income revealed a strong protective role of the latter within our cohort. Specifically, higher satisfaction with one’s socioeconomic situation correlates with a lower probability of developing this liver disease.

The observed correlation confirms that the onset of MASLD is influenced not only by metabolic factors but also depends by socioeconomic and demographic factors. This conclusion is supported by a growing body of literature that examines not just the medical history and living conditions of individuals but also their broader social context (7, 10).

The steady increase in cost of living, in recent years, may pose a significant threat to public health due to higher prices for healthy food such as vegetables and fruit, making it less affordable (10). In Italy, particularly in the southern regions, wage increases have not kept pace with these rising costs. Consequently, people’s purchasing power has diminished, leading to a notable decline in income satisfaction (11). Therefore, dissatisfaction among lower classes can result in greater food insecurity (10, 12–16, 32, 33) caused by financial struggles and limited access to affordable, healthy food (10). This situations often leads to an increased consumption of low-cost energy-dense foods (e.g., soft drinks, fast food, sugary snacks), which may elevate the risk of developing MASLD or related conditions such as T2DM, hypertension, or obesity (10).

This critical situation is supported by various health indicators within our cohort (BMI, rMED score, and kcal intake). These indicators all suggest that individuals who are more satisfied with their income tend to consume a healthier diet that closely aligns with the Mediterranean diet. Conversely, more dissatisfied individuals tend to have higher BMIs, approaching obesity (BMI = 29.13 kg/m^2^) (34), and tend to consume more daily calories.

A contrasting trend is observed among female subjects: women develop MASLD at a lower percentage than men (44.0%, 327 out of 417 women; 54.6%, 302 out of 553 men), despite the wage disparity mentioned earlier. This can be explained by the behavior reported in several studies (10, 32, 33), where mothers, especially those facing financial hardship, tend to restrict their own food intake to ensure their children have enough to eat.

Our results align with existing literature, confirming confirm the strong link between low income and the personal dissatisfaction that often accompanies it, which is linked to a higher probability of developing MASLD disease.

### 4.1 Strengths and limitations

One of the strengths of this study is the large number of participants from a cohort in southeastern Italy, where the food culture is strongly linked to the Mediterranean diet. This study is significant given the importance of the Mediterranean diet in combating MASLD disease. Additionally, few studies have been conducted on type of cohort.

Another strong point is the social impact. Our results highlight the importance of addressing socioeconomic factors in the prevention and management of MASLD, necessitating a comprehensive approach to promote equitable access to health care, and affordable access to healthy food.

The main limitation is the analyzed cohort, which is limited to a small town in Southern Italy. This limitation prevents the results from being generalised to populations with differing characteristics.

## 5 Conclusion

In conclusion, this study confirms that the development of MASLD is influenced not only by the clinical history and lifestyle of individuals, but also by socioeconomic factors, such as satisfaction with personal income. Given the current challenging economic climate, it is important to recognize that the growing wage disparity between social classes may pose a threat not only economic stability but also to public health.

## Data Availability

The datasets presented in this study can be found in online repositories. The names of the repository/repositories and accession number(s) can be found at: the original data presented in this study are openly available in FigShare at: https://doi.org/10.6084/m9.figshare.28677308.v3.

## References

[1] Cheemerla S Balakrishnan M. Global epidemiology of chronic liver disease. Clin Liver Dis. (2021) 17:365–70. doi: 10.1002/cld.1061, 34136143 PMC8177826

[2] Sepanlou SG Safiri S Bisignano C Ikuta KS Merat S Saberifiroozi M . The global, regional, and national burden of cirrhosis by cause in 195 countries and territories, 1990–2017: a systematic analysis for the global burden of disease study 2017. Lancet Gastroenterol Hepatol. (2020) 5:245–66. doi: 10.1016/S2468-1253(19)30349-8, 31981519 PMC7026710

[3] Miao L Targher G Byrne CD Cao Y-Y Zheng M-H. Current status and future trends of the global burden of MASLD. Trends Endocrinol Metabol. (2024) 35:697–707. doi: 10.1016/j.tem.2024.02.007, 38429161

[4] El-Kassas M Cabezas J Coz PI Zheng M-H Arab JP Awad A. Nonalcoholic fatty liver disease: current global burden. Semin Liver Dis. (2022) 42:401–12. doi: 10.1055/a-1862-9088, 35617968

[5] Chrysavgis L Cholongitas E. From NAFLD to MASLD: what does it mean? Expert Rev Gastroenterol Hepatol. (2024) 18:217–21. doi: 10.1080/17474124.2024.2374472, 38934451

[6] Chan W-K Chuah K-H Rajaram RB Lim L-L Ratnasingam J Vethakkan SR. Metabolic dysfunction-associated Steatotic liver disease (MASLD): a state-of-the-art review. J Obes Metab Syndr. (2023) 32:197–213. doi: 10.7570/jomes23052, 37700494 PMC10583766

[7] Donghia R Bonfiglio C Giannelli G Tatoli R. Impact of education on metabolic dysfunction-associated steatotic liver disease (MASLD): a southern Italy cohort-based study. JCM. (2025) 14:1950. doi: 10.3390/jcm14061950, 40142757 PMC11943323

[8] Lee EY Nguyen VH Cheung R Nguyen MH. Trends of chronic liver diseases by income level and socioeconomic factors in the United States: a population-based study. Aliment Pharmacol Ther. (2024) 60:1374–87. doi: 10.1111/apt.18242, 39238267

[9] Guo W Song Y. Association of poverty income ratio with metabolic dysfunction–associated steatotic liver disease and liver fibrosis among US population. TJG. (2025) 36:488–96. doi: 10.5152/tjg.2025.25021, 40820830 PMC12351293

[10] Bruening M MacLehose R Loth K Story M Neumark-Sztainer D. Feeding a family in a recession: food insecurity among Minnesota parents. Am J Public Health. (2012) 102:520–6. doi: 10.2105/AJPH.2011.300390, 22390517 PMC3349989

[11] Salvati L Tridico P. Real wages and productivity: a lesson from Italy, 1980-2023. Struct Chang Econ Dyn. (2025) 75:261–72. doi: 10.1016/j.strueco.2025.08.004

[12] Scheier LM. What is the hunger-obesity paradox? J Am Diet Assoc. (2005) 105:883–5. doi: 10.1016/j.jada.2005.04.013, 15942535

[13] Crawford PB Webb KL. Unraveling the paradox of concurrent food insecurity and obesity. Am J Prev Med. (2011) 40:274–5. doi: 10.1016/j.amepre.2010.11.003, 21238879

[14] Paik JM Duong S Zelber-Sagi S Lazarus JV Henry L Younossi ZM. Food insecurity, low household income, and low education level increase the risk of having metabolic dysfunction–associated fatty liver disease among adolescents in the United States. Am J Gastroenterol. (2024) 119:1089–101. doi: 10.14309/ajg.0000000000002749, 38477467

[15] Becerra MB Hassija CM Becerra BJ. Food insecurity is associated with unhealthy dietary practices among US veterans in California. Public Health Nutr. (2017) 20:2569–76. doi: 10.1017/S1368980016002147, 27571849 PMC10261359

[16] Huet C Rosol R Egeland GM. The prevalence of food insecurity is high and the diet quality poor in Inuit communities3. J Nutr. (2012) 142:541–7. doi: 10.3945/jn.111.149278 22323760

[17] Cozzolongo R Osella AR Elba S Petruzzi J Buongiorno G Giannuzzi V . Epidemiology of HCV infection in the general population: a survey in a southern Italian town. Am J Gastroenterol. (2009) 104:2740–6. doi: 10.1038/ajg.2009.428, 19638964

[18] UNESCO United Nations Educational, Scientific and Cultural Organization. "International standard Classification of education, ISCED 1997". In: Hoffmeyer-Zlotnik JHP Wolf C, editors. Advances in Cross-National Comparison. Boston, MA: Springer US (2003). p. 195–220.

[19] Ganzeboom HBG. A new international socio-economic index [ISEI] of occupational status for the international standard classification of occupation 2008 [ISCO-08] constructed with data from the issp 2002–2007; with an analysis of quality of occupational measurement in ISSP. Lisbon, Portugal: (2010).

[20] Sever P. New hypertension guidelines from the National Institute for health and clinical excellence and the British hypertension society. J Renin-Angiotensin-Aldosterone Syst. (2006) 7:61–3. doi: 10.3317/jraas.2006.011, 17083059

[21] Williams B Mancia G Spiering W Agabiti Rosei E Azizi M Burnier M . 2018 ESC/ESH guidelines for the management of arterial hypertension. Eur Heart J. (2018) 39:3021–104. doi: 10.1093/eurheartj/ehy339 30165516

[22] Riboli E Hunt K Slimani N Ferrari P Norat T Fahey M . European prospective investigation into cancer and nutrition (EPIC): study populations and data collection. Public Health Nutr. (2002) 5:1113–24. doi: 10.1079/PHN2002394, 12639222

[23] Riboli E. The EPIC project: rationale and study design. European prospective investigation into cancer and nutrition. Int J Epidemiol. (1997) 26:6S–14S. doi: 10.1093/ije/26.suppl_1.S6, 9126529

[24] Matthews DR Hosker JP Rudenski AS Naylor BA Treacher DF Turner RC. Homeostasis model assessment: insulin resistance?-cell function from fasting plasma glucose and insulin concentrations in man. Diabetologia. (1985) 28:412–9. doi: 10.1007/BF00280883, 3899825

[25] Chiloiro M Caruso MG Cisternino AM Inguaggiato R Reddavide R Bonfiglio C . Ultrasound evaluation and correlates of fatty liver disease: a population study in a Mediterranean area. Metab Syndr Relat Disord. (2013) 11:349–58. doi: 10.1089/met.2012.0169, 23758075

[26] Pisani P. Relative validity and reproducibility of a food frequency dietary questionnaire for use in the Italian EPIC centres. Int J Epidemiol. (1997) 26:152S–60S. doi: 10.1093/ije/26.suppl_1.S152, 9126543

[27] Dulai PS Singh S Patel J Soni M Prokop LJ Younossi Z . Increased risk of mortality by fibrosis stage in nonalcoholic fatty liver disease: systematic review and meta-analysis. Hepatology. (2017) 65:1557–65. doi: 10.1002/hep.29085, 28130788 PMC5397356

[28] Simon SD. Understanding the odds ratio and the relative risk. J Androl. (2001) 22:533–6. doi: 10.1002/j.1939-4640.2001.tb02212.x, 11451349

[29] Daneshvar A Mousa G. Regression shrinkage and selection via least quantile shrinkage and selection operator. PLoS One. (2023) 18:e0266267. doi: 10.1371/journal.pone.0266267, 36795659 PMC9934385

[30] Belsley DA Kuh E Welsch RE. Regression Diagnostics: Identifying Influential Data and Sources of Collinearity. 1st ed. Wiley (1980). doi: 10.1002/0471725153

[31] Alsemgeest L. Family communication about money: why the taboo? MJSS (2014). doi: 10.5901/mjss.2014.v5n16p516

[32] Wiig Dammann K Smith C. Factors affecting low-income women’s food choices and the perceived impact of dietary intake and socioeconomic status on their health and weight. J Nutr Educ Behav. (2009) 41:242–53. doi: 10.1016/j.jneb.2008.07.003, 19508929

[33] McIntyre L Glanville NT Raine KD Dayle JB Anderson B Battaglia N. Do low-income lone mothers compromise their nutrition to feed their children? CMAJ. (2003) 168.686–91. 12642423 PMC154913

[34] Expert Panel on the Identification, Evaluation, and Treatment of Overweight and Obesity in Adults (U.S.). Clinical guidelines on the identification, evaluation, and treatment of overweight and obesity in adults: the evidence report University of Minnesota: National Institutes of Health, National Heart, Lung, and Blood Institute, 1998. 2:51S–209S (2009). 9813653

