# Supplementary material for: Personal income perception and the risk of metabolic dysfunction-associated steatotic liver disease
Source: Front Nutr. 2026 Mar 5;13:1739165. doi: 10.3389/fnut.2026.1739165 (PMC12999383; doi:10.3389/fnut.2026.1739165)
Supplement: Supplementary file 1 [file Table_1.docx]

**SUPPLEMENTARY MATERIAL**

Supplementary Table 1. Qualitative ultrasound criteria for liver morphology evaluation.

| **Dimension** | Normal | Increased | Reduced |
| --- | --- | --- | --- |
| **Margins** | Regular | Irregulars | -- |
| **Ecostructure** | Homogeneous/Normoechogenic | Uneven | Hyperechogenic |

Supplementary Table 2. Ultrasound scoring system for the assessment of hepatic steatosis severity.

| **Contrast between liver and renal parenchyma** | **Ultrasound beam penetration** | **Vascular blurring (especially veins)** |
| --- | --- | --- |
| Homogeneous echogenicity with no evident liver–kidney contrast (0) | Hepatic parenchyma clearly visible from the surface to the diaphragm (0) | Vascular structures clearly visible (0) |
| Mild discrepancy in hepatic–renal echogenicity (1) | Attenuation of the ultrasound beam with partial loss of visualization of deep liver structures or diaphragm (1) | Loss of visualization of vascular structures (1) |
| Wide discrepancy between hepatic and renal (2) | Marked attenuation of the ultrasound beam with loss of visualization of deep liver structures and diaphragm (2) | Vascular structures poorly visible or not visible (2) |
| ***Steatosis Score****: Absent (0); Mild (1-2); Moderate (3-5); Severe (6)* | | |

Supplementary Table 3. Variance Inflation Factor

| **Variables** | **VIF** |
| --- | --- |
| Personal Assessment of Family Income |  |
| Totally Insufficient (Ref.) | -- |
| Just Sufficient | 5.96 |
| Sufficient | 7.94 |
| More than Sufficient | 3.21 |
| Good | 1.79 |
| Gender | 1.13 |
| Age (<65 yo vs >65 yo) | 1.47 |
| Personal Income | 1.12 |
| Marital Status | 1.08 |
| Work Status | 1.40 |
| House Location | 1.02 |
| Kcal intake (x day) | 1.12 |
| rMED Score | 1.07 |
| HOMA-IR | 1.03 |
| Mean VIF | 2.26 |

*Abbreviations: VIF, Variance Inflation Factor, rMED, Relative Mediterranean Diet,
HOMA-IR, homeostatic model assessment of insulin resistance*
